# *Akkermansia muciniphila* Modulates Central Nervous System Autoimmune Response and Cognitive Impairment by Inhibiting Hippocampal NLRP3‐Mediated Neuroinflammation

**DOI:** 10.1111/cns.70320

**Published:** 2025-03-06

**Authors:** Xiaobing Li, Dengna Lin, Xin Hu, Xiongwei Shi, Wenxuan Huang, Yi Ouyang, Xiaohong Chen, Yingqiong Xiong, Xiaomu Wu, Daojun Hong, Hao Chen

**Affiliations:** ^1^ Department of Neurology The First Affiliated Hospital, Jiangxi Medical College, Nanchang University Nanchang China; ^2^ Institute of Neurology, Jiangxi Academy of Clinical Medical Science, the First Affiliated Hospital, Jiangxi Medical College, Nanchang University Nanchang China; ^3^ Key Laboratory of Rare Neurological Diseases of Jiangxi Provincial Health Commission Jiangxi Medical College, Nanchang University Nanchang China; ^4^ Department of Gastroenterology The Sixth Affiliated Hospital, Sun Yat‐Sen University Guangzhou China; ^5^ Guangdong Institute of Gastroenterology Guangdong Provincial Key Laboratory of Colorectal and Pelvic Floor Diseases, Supported by National Key Clinical Discipline Guangzhou China; ^6^ Department of Neurology and Multiple Sclerosis Research Center The Third Affiliated Hospital, Sun Yat‐Sen University Guangzhou China

**Keywords:** *Akkermansia muciniphila*, cognitive impairment, EAE, multiple sclerosis, NLRP3

## Abstract

**Background:**

Numerous studies have demonstrated the significant role of *Akkermansia muciniphila* ( *A. muciniphila* ) in enhancing host immune responses and metabolic functions. However, its increased presence in multiple sclerosis (MS) patients has led to a focus on the relationships between *A. muciniphila* and diseases, with the underlying mechanisms remaining unknown.

**Method:**

Solochrome cyanin, hematoxylin–eosin staining (H&E) and immunofluorescence staining were used to assess demyelination and inflammation. Gut microbiota changes were examined by 16S rRNA sequencing. Intracellular cytokine levels were assessed by flow cytometry. Cognitive impairment was evaluated using four behavioral tests. Intestinal barrier function and pyrin domain‐containing protein 3 (NLRP3)‐mediated neuroinflammation were evaluated by immunoblotting.

**Results:**

We found that treatment with an appropriate dose of *A. muciniphila* (5.0 × 10^7^ CFU/mL) reduced neuropathology and disease severity in experimental autoimmune encephalomyelitis (EAE) mice. In addition, *A. muciniphila* supplementation increased the diversity and abundance of intestinal microbiota while decreasing the *Firmicutes/Bacteroidetes* ratio. Moreover, it improved intestinal barrier function and attenuated Th17 responses in the gut, central nervous system (CNS), and lymphoid tissues, without affecting Treg response in the lymphoid tissue. Furthermore, *A. muciniphila* administration partly regulated cognitive impairment and hippocampal NLRP3‐mediated neuroinflammation.

**Conclusion:**

Our results suggest that *A. muciniphila* holds promise as a probiotic for treating NLRP3‐associated inflammatory disorders and cognitive impairment, including MS.

## Graphical abstract

*A. muciniphila* may regulate neuroinflammation and cognitive impairment via hippocampal NLRP3‐mediated neuroinflammation, and the peripheral T cell response might modulate the relationship between intestinal microbiota and cognitive behavior.

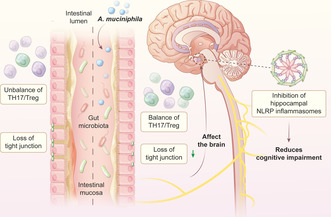

## 1 Introduction

Multiple sclerosis (MS) is the most widespread chronic inflammatory and neurodegenerative disorder affecting the CNS. Globally, about 2.8 million individuals (35.9/100,000) suffered from MS [1]. The etiology of MS is intricate and multifaceted, implicating a complex interplay of recognized genetic susceptibility factors, chiefly genes governing the immune system, alongside environmental influences such as smoking, insufficient vitamin D, lipoic acid and sun exposure, and infectious agents [2, 3]. Increasingly, the gut microbiota is being recognized as a pivotal susceptibility and protective element in MS. Accumulating evidence demonstrates the significance of gut‐to‐systemic immune function and metabolites in MS, suggesting that modulating the microbiome could hold therapeutic promise for these conditions [4].

Probiotics, encompassing bacteria and yeast, are living microorganisms that provide advantageous effects to the host when consumed in suitable amounts [5]. *Akkermansia muciniphila* ( *A. muciniphila* ), a gut symbiont thriving in the mucosal layer, emerges as a promising candidate among probiotics. In animal models, supplementation with *A. muciniphila* has shown promise in reducing obesity and its associated comorbidities, mitigating neurodegenerative disorders, and counteracting progeria [6, 7]. However, numerous studies have revealed an overrepresentation of *A. muciniphila* in patients afflicted with progressive and relapsing–remitting MS [4, 8]. In a previous study, we also reported an elevated prevalence of *A. muciniphila* in the stool metagenomic profiles of 40 autoimmune encephalitis patients [9]. Interventional research on *A. muciniphila* remains predominantly confined to animal experimentation, with limited investigations into its efficacy and safety in humans. Hence, there is a pressing need for studies elucidating the bacteriological characteristics, safety profile, and pathogenicity of *A. muciniphila* .

Dysregulation in the differentiation of Th17 and Treg cells, alongside imbalances in intestinal flora and damage to the gut mucosal barrier, constitutes crucial links in the onset and progression of autoimmune diseases [9]. Treg cells play a fundamental role in maintaining immune homeostasis and inhibiting autoimmunity in MS/EAE by suppressing the activation of other immune cell types. Th17 cells and Th1 are thought to promote CNS autoimmunity and are strongly associated with disease activity and CNS dysfunction. Treg cells and Th17 cells can convert into each other, and the Treg/Th17 balance is vital to the immunity of intestinal mucosal barriers and the inflammatory responses in MS [10]. Moreover, the NLRP3 inflammasome‐related molecules are involved in the pathogenesis of MS. It is comprised of the NLRP3, apoptosis‐associated speck‐like protein containing a carboxyterminal CARD (ASC), and pro‐caspase‐1. Research has documented upregulated expression levels of NLRP3 and IL1β genes in MS plaques, along with elevated serum levels of IL18, caspase1, and ASC in MS patients [11]. The NLRP3‐mediated inflammasome needs nuclear transcription factor‐κB (NF‐κB) signaling pathway activation. Once the NF‐κB signaling pathway is activated, it leads to the upregulated transcription of inflammasome‐related components, such as NLRP3, pro‐IL‐1β, and pro‐IL‐18. Then the inflammasome‐adaptor protein ASC is recruited to NLRP3 and interacts with caspase‐1, leading to its activation. Activated caspase‐1 can catalyze the maturation of pro‐inflammatory cytokines IL‐1β and IL‐18 [12]. Furthermore, gut microbiota from NLRP3^−/−^ mice exhibit a notable increase in *Firmicutes* alongside a decrease in *Bacteroidetes* [13]. Additionally, NLRP3 gene (NLRP3R258W) mice exhibit diminished levels of *A. muciniphila* and *Prevotella*, which are considered colitogenic bacteria [14]. This underscores a significant link between gut microbiota and NLRP3 in MS pathogenesis. The intestinal microbiota directly impacts the integrity of the gut barrier and the function of immune cells in gut‐associated lymphoid tissues, thereby influencing both local and systemic immune responses [15]. Occludin, Claudin‐5, E‐Cadherin, and JAM‐A are known to play a crucial role in maintaining the physical barrier function of the epithelium [16]. Moreover, cognitive impairments and anxiety can manifest in the early stages of MS, even in the absence of other neurological deficits [17]. The intestinal microbiota can influence the CNS through various pathways and contribute to the development of cognitive disorders [18]. However, whether gut microbiota modulates CNS autoimmunity and cognitive impairment by influencing NLRP3 inflammasome‐related molecules in MS remains unclear.

To address this gap, we conducted a study utilizing *A. muciniphila* in experimental autoimmune encephalomyelitis (EAE) mice, an established animal model for human MS. Initially, we demonstrated that *A. muciniphila* alleviated symptoms in EAE mice and assessed its clinical and neuropathological effects. Different doses of *A. muciniphila* were administered to mice in the EAE group for 3 weeks to determine the optimal dosage. The selection of *A. muciniphila* concentrations was based on prior animal studies and our initial dose exploration experiment with *C. butyricum* (a probiotic) [19]. Subsequently, we employed 16S rRNA gene sequencing to observe changes in the gut microbiome. Additionally, we evaluated changes in behavior tests, intestinal barrier function, and T cell dynamics in the different organizations, noting the decrease and increase in Th17 response and regulatory T cells, respectively. Dysbiosis in the gut may contribute to inflammatory processes both peripherally and centrally, potentially leading to cognitive deficits [20]. To explore the potential link between gut microbiota modulation with *A. muciniphila* and relevant behavioral phenotypes, we also conducted a behavioral study. Finally, we discovered that *A. muciniphila* could suppress NLRP3 inflammasome activation by triggering pyroptosis in the hippocampal region of EAE mice. Our findings suggest that *A. muciniphila* may modulate neuroinflammation and cognitive impairment via hippocampal NLRP3‐mediated neuroinflammation, and peripheral T cell response might modulate the relationship between intestinal microbiota and cognitive behavior in EAE mice.

## 2 Materials and Methods

### 2.1 Animals and Reagents

Considering that female mice are more susceptible to EAE modeling [21], four‐ to six‐week‐old female specific pathogen‐free animals (C57BL/6J WT mice, *n* = 20/group) were supplied by Guangdong Medical Laboratory Animal Center (Guangzhou, China). We divided the experimental process into four time periods: T1 (Basetime), T2 (Immunization), T3 (EAE peak time), T4 (EAE recovery time), T5 (hippocampal neuroinflammation detection). The mice were allowed to acclimate in the laboratory for 7 days before starting the experiment. All experimental protocols adhered to the guidelines outlined in the National Institutes of Health Guide for Care and Use of Laboratory Animals and were approved by the Bioethics Committee of the First Affiliated Hospital Nanchang University (Approval ID: 2021‐9024). *A. muciniphila* in the form of freeze‐dried lysed cells (GDBIO1501, GDBIO‐TECH Biotechnology) was kept at −20°C. Here we introduce the EAE model based on C57BL/6J mice, which is generated by injection of myelin oligodendrocyte glycoprotein 35–55 (MOG 35–55), (CL. BioScientific LTD, Xi'an, China) as an antigen. The amino acid sequences were verified through mass spectroscopy and amino acid analyses, with the peptide purity exceeding 95%. *Mycobacterium tuberculosis* H37RA was acquired from Difco (Detroit, MI, USA), while Pertussis toxin (PTX) was procured from Alexis Corp (CA, USA). Fluorescein isothiocyanate (FITC)‐conjugated anti‐mouse CD4, APC‐conjugated anti‐mouse CD25, BV421‐conjugated anti‐mouse IL17A, P‐phycoerythrin (PE)‐conjugated anti‐mouse granulocyte‐macrophage colony‐stimulating factor (GM‐CSF), and PE‐conjugated anti‐mouse Foxp3 were sourced from BioLegend (CA, USA). Occludin, claudin‐5, e‐cadherin, Junctional adhesion molecule A (JAM‐A), ASC, cleaved caspase 1, IL‐18, nuclear transcription factor‐κB (NF‐κB), p‐nuclear transcription factor‐κB (p‐NF‐κB) and NLRP3 were supplied by Cell Signaling Technology (USA).

### 2.2 Dose Optimization and Treatment Regimen

The animals were randomly divided into five groups with gavage administration: purified water‐treated mice, purified water‐treated EAE mice, *A. muciniphila* ‐treated (5.0 × 10^6^ CFU/mL), *A. muciniphila* ‐treated (5.0 × 10^7^ CFU/mL), and *A. muciniphila* ‐treated (5.0 × 10^8^ CFU/mL) EAE mice. Considering that the 3–4 week (weaning) period is optimal for probiotic intervention [22], three different concentrations of *A. muciniphila* in purified water (10 mL/kg) were administered intragastrically every day for 3 weeks prior to EAE induction.

### 2.3 Induction and Evaluation of EAE Through MOG35–55 Peptides

EAE induction followed established procedures [19, 23]. Briefly, female mice aged 7–9 weeks received an inguinal region subcutaneous injection of 300 μg MOG35‐55 peptide/animal emulsified in complete freund's adjuvant (CFA) containing 500 μg *Mycobacterium tuberculosis* H37RA. Subsequently, and once more 48 h later, the animals were administered an intraperitoneal injection containing 300 ng pertussis toxin (PTX) dissolved in 100 μL PBS. Seven days later, another injection of MOG35‐55 peptide in CFA was administered. Daily monitoring of the animals for disability was conducted, and EAE scores were assessed using a scale ranging from 0 to 5, as previously outlined [24].

### 2.4 Histological and Immunohistochemistry Assessment

At 21 days post‐immunization, the treated mice were subjected to paraformaldehyde (4%) for fixation, and the lumbosacral spinal cord was harvested, followed by embedding in paraffin. The paraffin section was subjected to solochrome cyanin and H&E staining to assess demyelination and inflammation, respectively. The inflammation was graded on the following scale [25]: 0, absence of inflammatory cells; 1, presence of a few scattered inflammatory cells; 2, organization of inflammatory infiltrates around blood vessels; 3, extensive perivascular cuffing with or without parenchymal infiltration. Demyelination in the spinal cord was graded based on the scale outlined in previous literature [26]: 1, minimal subpial demyelination; 2, pronounced perivascular and subpial demyelination; 3, confluent subpial and perivascular demyelination; 4, extensive subpial and perivascular demyelination affecting half of the SC with infiltration of cellular components into CNS parenchyma; 5, widespread subpial and perivascular demyelination involving the entire cross‐section of the cord with infiltration of cellular elements into CNS parenchyma. Antibodies to myelin basic protein (MBP; at 1:200; Abcam) and non‐phosphorylated neurofilaments (clone SMI‐32, at 1:200; Sternberger Monoclonals) were used to double stain the demyelinated axons and injured axons and analyzed with the positive‐staining percentage (number of positive pixels/1 mm^2^).

### 2.5 Immunoblotting

For the investigation of protein levels of occludin, claudin‐5, e‐cadherin, JAM‐A in the gut, as well as ASC, Cleaved Caspase 1, IL‐18, NF‐κB, p‐NF‐κB and NLRP3 in the hippocampus of mice from different treatment groups (*n* = 6), Western blot analysis was conducted. Samples from the entire gut (Day 21 after the first immunization) and entire hippocampus (Day 50 after the first immunization) of mice receiving various treatments were loaded onto 10% SDS‐PAGE (20 mg protein/lane). The proteins were then transferred onto PVDF membranes (Bio‐Rad) and subsequently blocked with 5% non‐fat milk. Following this, the membranes were incubated overnight with primary antibodies targeting occludin (1:1000), claudin‐5 (1:1000), e‐cadherin (1:1000), JAM‐A (1:1000), ASC (1:1000), cleaved caspase 1 (1:1000), IL‐18 (1:1000), NF‐κB (1:1000), p‐NF‐κB (1:1000) and NLRP3 (1:1000). After rinsing thrice with TBST buffer, the membranes were exposed to anti‐mouse HRP and goat anti‐rabbit HRP for 30 min each. GAPDH served as the internal control.

### 2.6 Flow Cytometry

To assess intracellular cytokine levels, lymphocytes were extracted from peripheral lymph nodes (LNs), brain, spinal cord (SC), and small intestine lamina propria (SILP) 3 weeks post‐immunization as described previously [19]. These cells were subjected to stimulation, fixing, and permeabilization, followed by staining with fluorescent‐conjugated intracellular cytokine antibodies. The Foxp3‐Staining‐Buffer‐Set (BioLegend) was employed for intranuclear Foxp3 staining.

### 2.7 Behavioral Tests

The detection order of the four behavioral tests was elevated plus maze test—open‐field test—novel object recognition test—Morris water maze test [9].

#### 2.7.1 Elevated Plus Maze Test

The elevated plus maze consisted of two open arms (30 × 5 cm) and two closed arms (30 × 5 cm) with 15‐cm high walls. The arms extended from a center square platform (5 × 5 cm) and were arranged so that those of the same type were opposite each other. The apparatus was elevated to 50 cm above the floor. A behavioral test was conducted under the light of a 25‐W incandescent lamp. At the start of the trial, each mouse was placed on the center square platform and was allowed to move freely for 5 min. All sessions were automatically recorded with a computer‐based video tracking system.

#### 2.7.2 Open Field Test

The open field apparatus was a square field (length 50 cm, width 50 cm, height 40 cm) with the floor marked out into 16 sectors under the light of a 60‐W incandescent lamp. Each mouse was placed in the center of the enclosed area and was allowed to move freely for 3 min as the mouse has the strongest desire to explore a novel environment just within the early minutes when it entered the open field. During this period, behaviors were recorded, including grid crossing, rearing (rearing up on haunches with forelimbs 3–4 cm off the floor), and grooming. Each mouse was tested on the open field task just once. The open field was cleaned with alcohol after each animal to remove odors of the previous animal. The number of grid crossings was evaluated as an index of spontaneity or locomotor activity, and the frequency of rearing was evaluated as an index of exploratory behavior. Grooming is shown by animals in a situation of conflict and is in contrast to locomotor and rearing behaviors. The time spent in the central four sectors of the arena was recorded as the central part time. All sessions were automatically recorded with a computer‐based video tracking system.

#### 2.7.3 Novel Object Recognition Test

The novel object recognition (NOR) test is commonly used to evaluate non‐spatial memory. The experiment was performed in a 72 × 72 × 35 cm^3^ white open‐field box. The day before testing, mice were habituated to freely explore the experimental apparatus for 5 min without objects. Mice were then given a session of two trials with an inter‐trial interval of approximately 1 h. In the first trial, mice were placed into the experimental apparatus and allowed to explore two identical objects located equidistantly and symmetrically on the diagonal of the box for 5 min. During the second trial, mice were put into the box again; however, one of the familiar objects was replaced by a novel object with different colors and shapes; the mice were left in the apparatus for 5 min. The box and objects were carefully cleaned with 70% ethanol after each trial to clear the smell of mice. A camera was installed directly above the experimental apparatus to record the time that animals explored familiar objects and novel objects. The discrimination ratio was calculated as *N*/(*N* + *F*) (novel objects/novel objects + familial objects) × 100% to compare differences among groups.

#### 2.7.4 Morris Water Maze Test

The Morris water maze (MWM) was used to evaluate the spatial learning and memory of each group after the NOR test. The device consisted of a 150‐cm‐diameter round pool filled with water to a depth of 35 cm. The water temperature was maintained at 22°C ± 2°C. In the target quadrant (QIII) of the pool, a 15‐cm‐diameter escape platform was placed approximately 2 cm below the water surface. The different groups of mice first received a place navigation test for five consecutive days. Mice were gently put into the water and released facing the wall from one of four quadrants in a random order. They were allowed to find the escape platform for 60 s, and the latency to escape onto the platform was recorded. Mice were trained four times a day, with inter‐trial intervals of approximately 20 min. The escape latency was measured and analyzed. The day after the place navigation test, a 60‐s spatial probe test was conducted with the platform removed. The times of mice crossing the platform area and the dwell time in the target quadrant where the platform was located before were recorded during the probe test.

### 2.8 16S rDNA PCR and Sequencing Analysis

Stool specimens were harvested 3 weeks before immunization, and 16S rRNA sequencing was carried out following previously described procedures [19, 27]. Fecal specimens were promptly frozen at −80°C. Bacterial DNA extraction from fecal specimens was performed using the QIAamp‐DNA‐Stool‐Mini‐Kit (Qiagen, Germany). PCR conditions were as follows: initial denaturation at 98°C for 3 min, followed by 30 cycles of 98°C for 45 s, 55°C for 45 s, 72°C for 45 s, and a final extension at 72°C for 7 min. PCR products were purified with AmpureXP‐beads (AGENCOURT) to eliminate nonspecific products. The final library was quantified by determining the average molecular length using an Agilent‐2100‐Bioanalyzer (Agilent‐DNA‐1000‐Reagents) and quantifying the library through RT‐PCR (EvaGreen). The qualified library was subjected to paired‐end sequencing on a MiSeq system, employing either PE300 (PE301 + 8 + 8 + 301) or PE250 (PE251 + 8 + 8 + 251) (MiSeq‐Reagent Kit).

### 2.9 Statistical Analyses

All data were presented as means ± standard error of mean (SEM). Two‐tailed Student's *t*‐test or the non‐parametric Mann–Whitney test was performed to analyze the differences between the two groups. Non‐parametric data were analyzed using the Kruskal–Wallis test. For statistical analysis of sequencing data, R v2.15.3 and various indices tools including Shannon, Simpson, Chao 1, Observed Species, and ACE were utilized. Linear discriminant analysis effect size (LEfSe) analysis was employed to identify pathways or taxa exhibiting differential abundance between the naive group and the *A. muciniphila* group. This approach initially employs the nonparametric factorial Kruskal–Wallis sum‐rank test to determine features with significant differential abundances, followed by Linear Discriminant Analysis (LDA) to measure their effect sizes. Furthermore, metagenomes of the intestinal microbiome were predicted from 16S rRNA sequences using PICRUSt [28]. Statistical differences were considered significant at the *p* < 0.05 level and indicated with asterisks (*p < 0.05, **p < 0.01, ***p < 0.001, and ****p < 0.0001).

## 3 Results

### 3.1 *A. muciniphila* Attenuates Inflammatory Demyelination in the EAE Mouse Model

**FIGURE 1:**
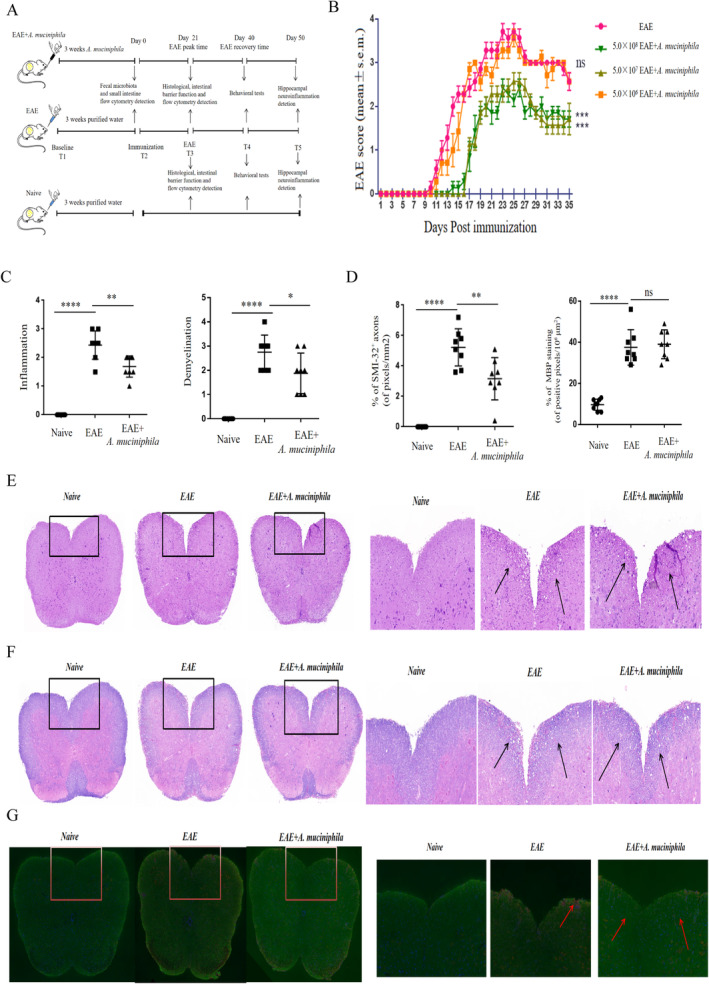
*A. muciniphila* attenuates the neuropathology and clinical severity of EAE mice at T3. (A) Schematic representation of the experimental timeline at baseline. (B) Daily assessment of EAE clinical scores in a representative experiment (*n* = 8). (C and D) Quantitative analysis of axonal damage, MBP staining, demyelination and inflammation in the lumbosacral spinal cord of the two groups (*n* = 8/group). Each data point denotes an individual mouse, and each bar represents mean ± SEM. *p < 0.01, **p < 0.001, ****p < 0.0001, ns, not significant. (E, F, and G) Histopathological assessment (*n* = 8): Lumbosacral spinal cord were isolated at 21 days post‐immunization and subjected to H&E staining, solochrome cyanin impregnation, or immunostaining for myelin basic protein (MBP; in green) and SMI‐32^+^ damaged axons (in red). Scale bar: 200 μm.

The experimental timeline and specimen collection are outlined in Figure 1A. It was observed that a dose of 5.0 × 10^6^ CFU/mL *A. muciniphila* treatment did not confer a protective effect on EAE mice. However, both 5.0 × 10^7^ and 5.0 × 10^8^ CFU/mL doses exhibited reduced morbidity and symptom severity compared to the EAE group, with no obvious difference between the two doses, as indicated by reduced disease scores (Figure 1B). Thus, 5.0 × 10^7^ CFU/mL was chosen as the optimal dosage for subsequent experiments. Neuropathological analysis revealed that the *A. muciniphila* group exhibited reduced demyelination and inflammation in the lumbar SC, as assessed by solochrome cyanin and H&E staining (Figure 1C,E,F). Additionally, there was a reduction in SMI‐32^+^ damaged axons, and no significant difference in MBP staining in the lumbar SC of the *A. muciniphila* group (Figure 1D,G). Importantly, no obvious adverse effects of *A. muciniphila* were observed at the selected dosage.

### 3.2 *A. muciniphila* Promotes Microbial Diversity and Induces Changes in the Compositions of the Gut Microbiome

**FIGURE 2:**
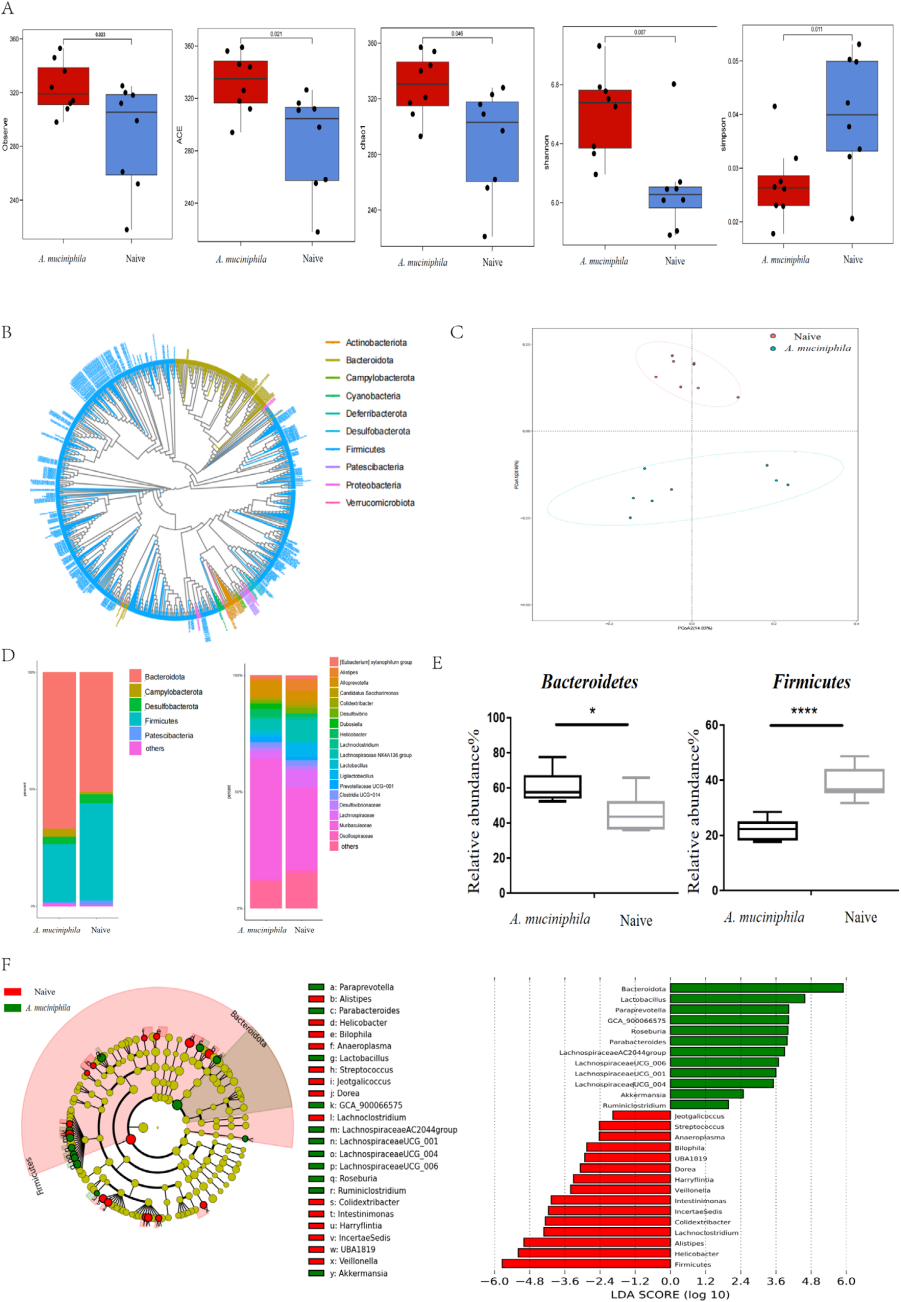
*A. muciniphila* induces alterations in gut microbiota composition in EAE mice before immunization. (A) Significant changes are observed in the quantity of Shannon diversity index, OTUs, ACE, Chao1, and Simpson index values between the two groups (*n* = 8). Mean ± SEM.(B) Phylogenetic tree depicting genus‐level species relationships. (C) Principal‐coordinate analysis of Bray–Curtis dissimilarity demonstrating microbiome similarity between the two groups. (D) Relative abundances of intestinal at genus and phylum levels. (E) Notable alterations in bacterial phylum‐level relative abundances between the two groups. *p < 0.01, ****p < 0.0001. (F) Identifying differential microbiota in *A. muciniphila* ‐treated mice using the LDA and LEfSe pipelines. Cladogram generated via the LEfSe approach depicts the phylogenetic distributions of intestinal microbiota in the two groups. LDA scores highlight obvious bacterial distinctions between the groups.

We utilized 16S rRNA sequencing targeting the V3 and V4 regions to investigate alterations in the gut microbiota. Our analysis revealed that the administration of *A. muciniphila* led to a significant enhancement in both diversity and abundance of the gut microbiota, as evidenced by increased index values for observed species (*p* = 0.023), ACE (*p* = 0.021), Chao 1 (*p* = 0.046), and Shannon (*p* = 0.007), accompanied by a decrease in the Simpson index value (*p* = 0.011) (Figure 2A). The phylogenetic tree depicted the interrelations among intestinal flora compositions in mice (Figure 2B). Notably, principal component analysis demonstrated a clear separation in the clustering of intestinal microbiota composition between the *A. muciniphila* ‐treated mice and the ad libitum group (Figure 2C). At the phylum level, we observed a decrease and an increase in the abundances of *Firmicutes* and *Bacteroidetes*, respectively, thus leading to a decreased *Firmicutes*/*Bacteroidetes* (F/B) ratio in *A. muciniphila* ‐treated mice. Genus‐level analysis revealed a decrease in *Desulfovibrionaceae*, *Lachnospiraceae*, and *Alistipes*, along with an increase in *Prevotellaceae UCG‐001* in *A. muciniphila* ‐treated mice (Figure 2D). Additionally, Figure 2E illustrates an obvious increase in *Bacteroidetes* and a decline in *Firmicutes* following *A. muciniphila* treatment at the phylum level. Differential gut microbiota analysis using LEfSe methods (Figure 2F) identified an increased abundance of *Paraprevotella*, *Ruminiclostridium*, and *Roseburia*, while *Streptococcus* abundance decreased with *A. muciniphila* treatment, as indicated by the LDA scores.

### 3.3 *A. muciniphila* Inhibits the Pathogenic T Cell Differentiation in EAE Mice

**FIGURE 3:**
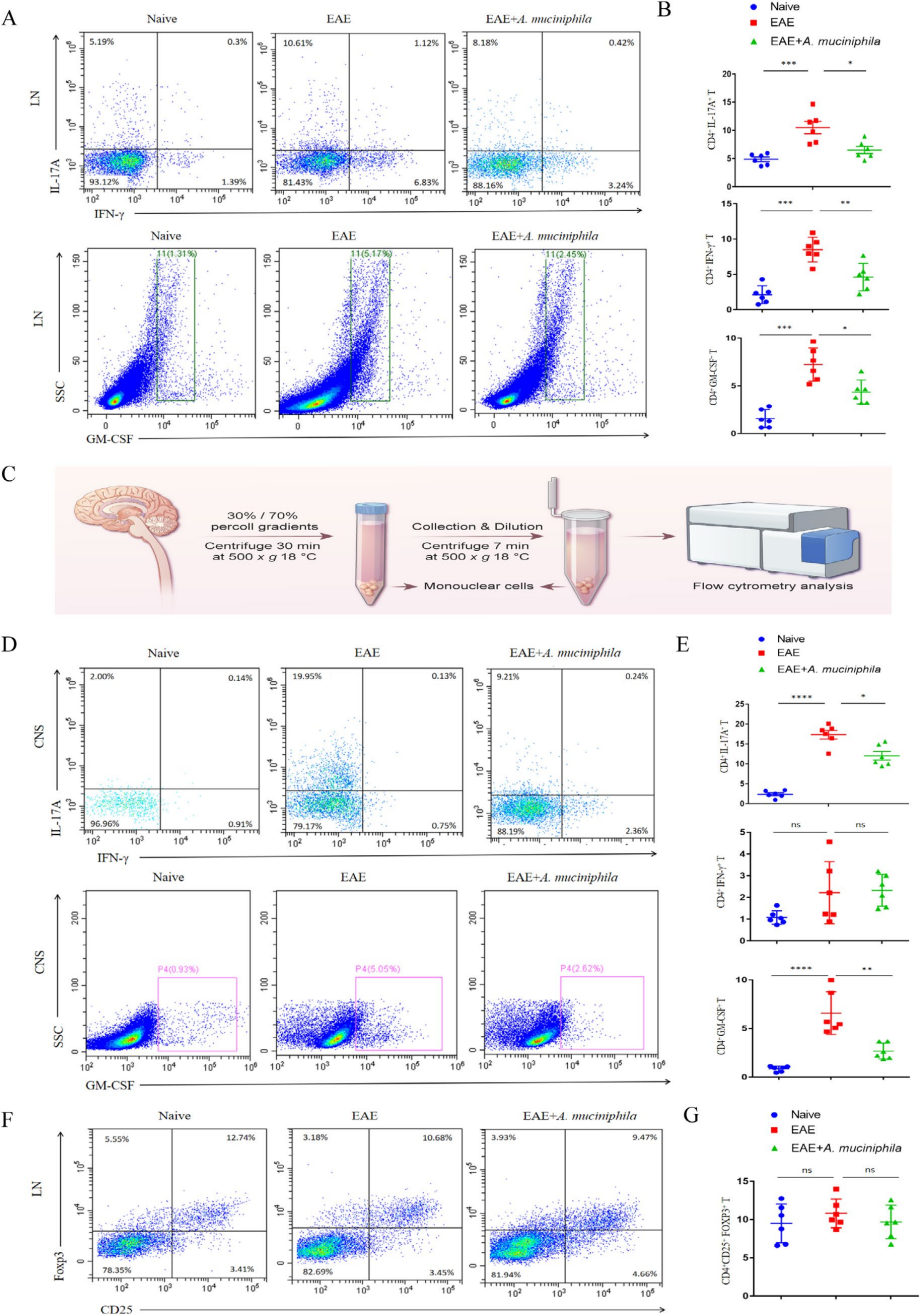
*A. muciniphila* suppresses pathogenic T cell differentiation in EAE mice (*n* = 6). (A and B) Analysis of subpopulations of differentiated Th1/Th17 cells in peripheral LNs via intracellular staining for GM‐CSF, IL17A, and IFN‐γ. (C) Collection of mononuclear cells from the SC and brain using 70%/30% percoll gradients. (D and E) Analysis of subpopulations of differentiated Th1/Th17 cells in the CNS via intracellular staining for GM‐CSF, IL17A, and IFN‐γ. (F and G) Analysis of subpopulations of Treg cells differentiated in peripheral LNs via intracellular staining for FOXP3. Mean ± SEM. *p < 0.05, **p < 0.01, ***p < 0.001, ****p < 0.0001, ns, not significant.

Given that activated CD4^+^ T cells are key contributors to EAE pathogenesis [29], we conducted a detailed analysis of CD4^+^ Th cell subsets in the peripheral LNs and CNS of EAE mice using flow cytometry. After lymph node grinding to obtain single‐cell suspension, we performed cell surface and intracellular staining following restimulation and secretion blocking. Notably, the administration of *A. muciniphila* significantly impeded Th1/Th17 differentiation, as indicated by a decrease in the proportions of CD4^+^ GM‐CSF^+^ and CD4^+^ IL17A^+^ cell populations (Figure 3A,B). Subsequently, we examined Th phenotypes in the SC and brain using the 70%/30% percoll method (Figure 3C). Remarkably, *A. muciniphila* administration led to a notable reduction in CD4^+^ T cell infiltration and pathological Th17 differentiation in the CNS, attributed to the suppression of GM‐CSF and IL17A secretion, while there was no marked alteration in CD4^+^ IFN‐γ^+^ cell populations (Figure 3D,E). Generally speaking, regulatory T cells were enumerated using CD4^+^, CD25,^+^ and Foxp3^+^ markers by flow cytometry [19]. However, no obvious changes were detected in the proportion of regulatory T cells (CD4^+^CD25^+^FOXP3^+^T cells) secretion from inguinal LNs in EAE mice (Figure 3F,G).

### 3.4 *A. muciniphila* Alters Intestinal Barrier Function and T Cell Differentiation in the Intestines

**FIGURE 4:**
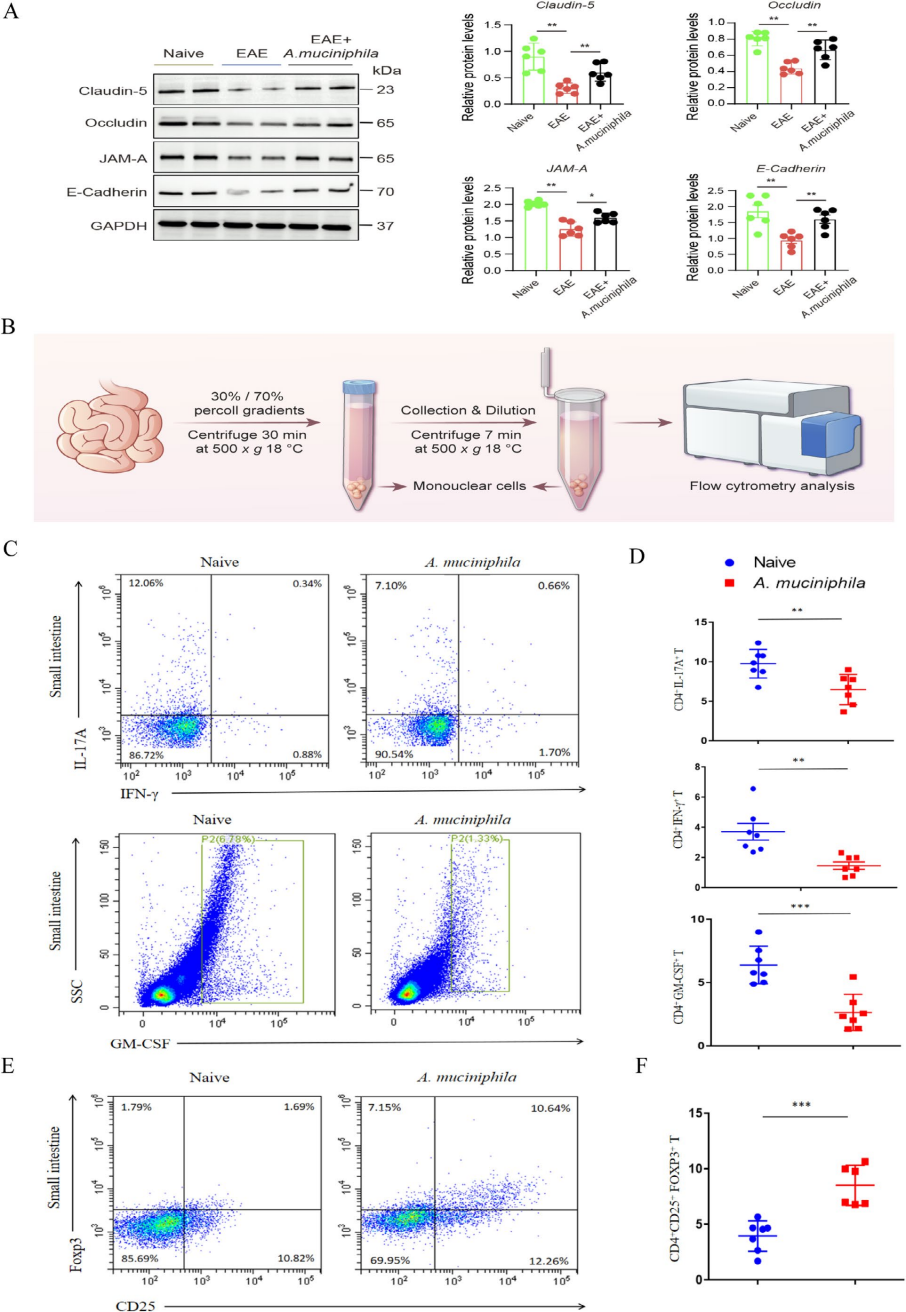
*A. muciniphila* treatment alters intestinal barrier function and T cell differentiation in the gut after 3 weeks at T2. (A) Western blot and densitometric analysis showing the expression of tight junction proteins (Occludin, Claudin‐5, E‐Cadherin, JAM‐A) in ileal tissue (*n* = 6). (B) Collection of mononuclear cells from the small intestine using 70%/30% percoll gradients. (C and D) Analysis of subpopulations of differentiated Th1/Th17 cells in the small intestine via intracellular staining for GM‐CSF, IL17A, and IFN‐γ (*n* = 7). (E and F) Analysis of subpopulations of Treg cells differentiated in the small intestine via intracellular staining for FOXP3. Mean ± SEM. *p < 0.05, **p < 0.01, ***p < 0.001.

We found remarkable differences in the structure and function of the intestinal barrier, including changes in intestinal permeability and the expression of tight junction proteins, particularly in the ileum tissue (Figure 4A). Furthermore, we investigated the pathogenic Th and Treg phenotypes in the SILP using the 70%/30% percoll method (Figure 4B). Additionally, we assessed the levels of Th17 and Treg differentiation in the SILP following 3 weeks of *A. muciniphila* treatment. Consistent with the changes in the microbial communities, we observed decreased production of IL17A, IFN‐γ, and GM‐CSF in the SILP of *A. muciniphila* ‐treated mice (Figure 4C,D). Moreover, *A. muciniphila* treatment promoted Treg differentiation, as evidenced by increased secretion of CD4^+^CD25^+^FOXP3^+^T cells in EAE mice (Figure 4E,F).

### 3.5 *A. muciniphila* Treatment Partly Prevents Cognitive Impairment in EAE Mice

**FIGURE 5:**
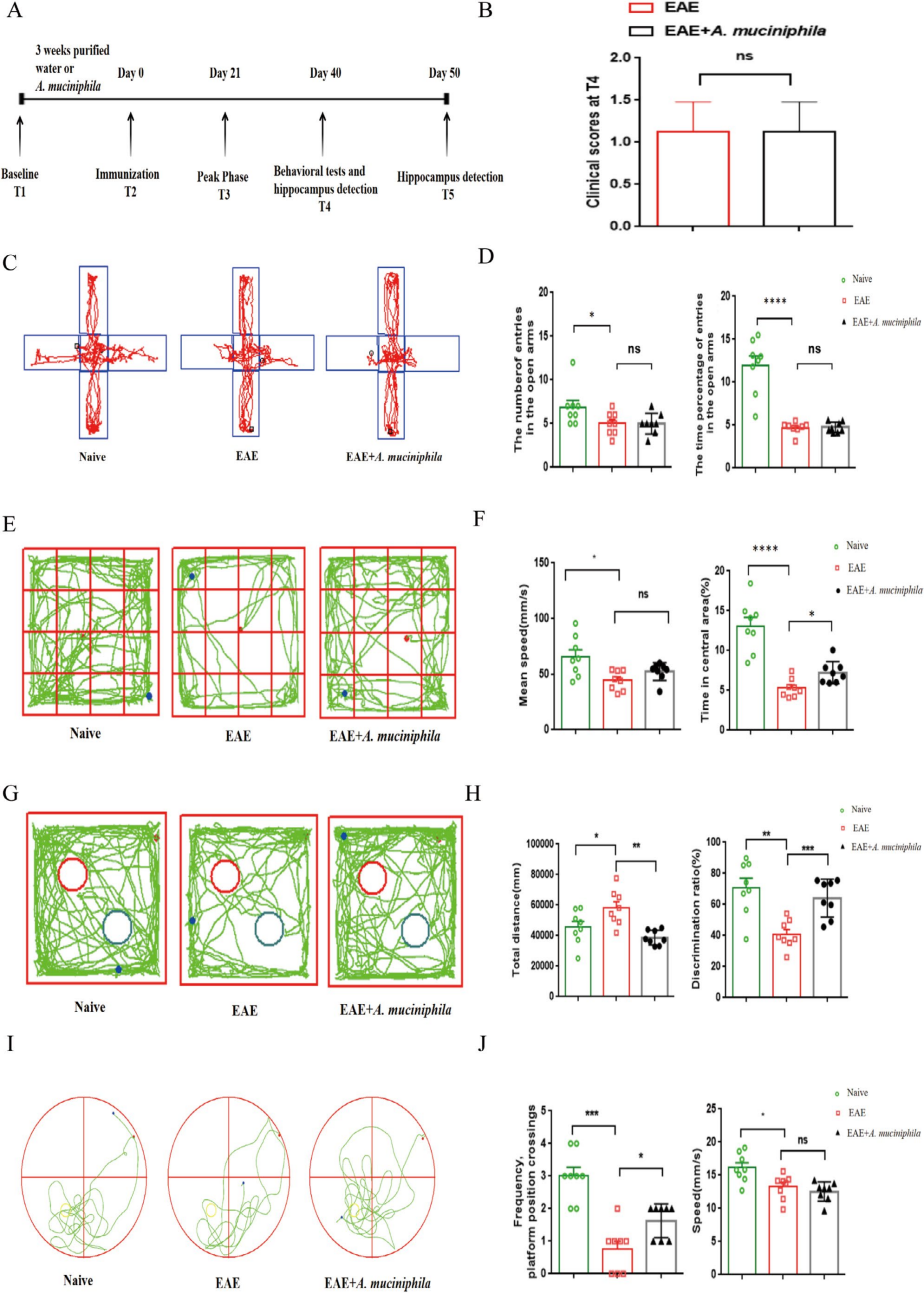
*A. muciniphila* treatment alleviates cognitive impairment in EAE mice. (A) Schematic representation of the behavioral tests. (B) EAE clinical scores at T4 demonstrated no obvious difference between the EAE and *A. muciniphila* group (*n* = 8). (C and D) Elevated‐plus‐maze test. Compared to naive mice, EAE mice exhibited a decreased number of entries and time percentage of entries. No obvious difference was found between the EAE and *A. muciniphila* group. (E and F) Open‐field test. Compared to naive mice, EAE mice exhibited decreased distance, mean speed, and spent a shorter time in the center area. Although no obvious difference was found in mean speed between the EAE and *A. muciniphila* group, *A. muciniphila* ‐treated mice spent obviously more time in the center area. (G and H) Novel object recognition test. Compared to EAE mice, *A. muciniphila* ‐treated mice displayed an increased discrimination ratio and decreased total distance traveled. (I and J) Morris water maze tests. Compared to naive mice, EAE mice exhibited decreased platform position crossings and mean speed. No obvious difference was found in speed between the EAE and *A. muciniphila* group, but *A. muciniphila* ‐treated mice exhibited remarkably more platform position crossings than EAE mice. Mean ± SEM. *p < 0.05, **p < 0.01, ***p < 0.001, ***p < 0.0001, ns, not significant.

The experimental protocol for behavioral assessments is depicted in Figure 5A. To minimize behavioral interference during the disease onset, behavioral experiments were conducted at T3, where the EAE clinical scores demonstrated no obvious difference between the two EAE groups (Figure 5B). In the elevated‐plus‐maze test, there were no differences in the number of entries and time percentage of entries covered in the open arms between the EAE and *A. muciniphila* groups. Compared to naive mice, EAE mice exhibited a decreased number of entries and time percentage of entries (Figure 5C,D). In the open‐field test, mice treated with *A. muciniphila* exhibited no hyperactivity (total distance and mean speed) but spent a longer time in the center area between the EAE and *A. muciniphila* groups. Compared to naive mice, EAE mice exhibited decreased distance, mean speed, and spent a shorter time in the center area (Figure 5E,F and Data S1). These two behavioral tests indicate no obvious anti‐anxiety effects of *A. muciniphila* . Cognitive behaviors were assessed through NOR and Morris‐water‐maze tests. In the Morris‐water‐maze tests, *A. muciniphila* ‐treated mice demonstrated significantly more platform position crossings than EAE mice. Compared to naive mice, EAE mice exhibited decreased platform position crossings and mean speed, indicating an improvement in the *A. muciniphila* group regarding cognitive difficulties affecting spatial orientation and memory acquisition (Figure 5I,J). Additionally, compared to the EAE mice, *A. muciniphila* ‐treated mice exhibited a decreased total distance traveled and an increased discrimination ratio and index in the NOR test, suggesting a significant amelioration of memory‐learning deficits in EAE mice following *A. muciniphila* treatment (Figure 5G,H and Data S1). Overall, these four behavioral tests collectively indicate that *A. muciniphila* treatment may exert no obvious anti‐anxiety effects and partly improve cognitive impairment.

### 3.6 *A. muciniphila* Treatment Prevents Hippocampal NLRP3‐Mediated Neuroinflammation in EAE Mice

**FIGURE 6:**
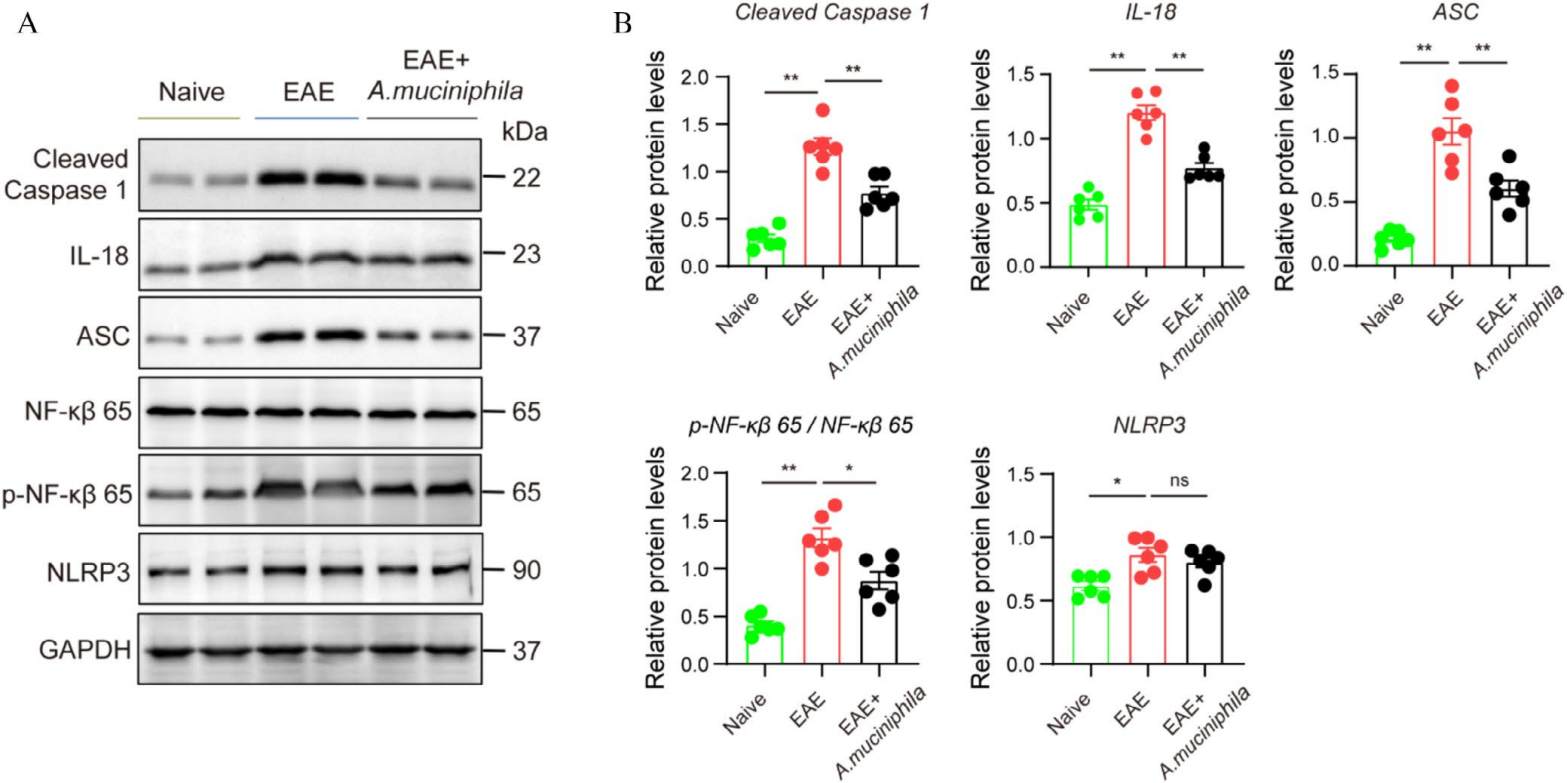
*A. muciniphila* treatment alleviates hippocampal NLRP3‐mediated neuroinflammation in EAE mice. (A and B) Immunoblotting and densitometric analysis showing the expression of ASC, Cleaved Caspase 1, IL‐18, NF‐κB, p‐NF‐κB and NLRP3 in the three groups. (*n* = 6) Mean ± SEM. *p < 0.05, **p < 0.01, ns, not significant.

The NLRP3 inflammasome plays a crucial role in regulating the microbiota–gut–brain axis [30]. To ascertain whether treatment with *A. muciniphila* prevented cognitive impairment by inhibiting hippocampal NLRP3‐mediated neuroinflammation activation, we assessed the expression levels of ASC, Cleaved Caspase 1, IL‐18, NF‐κB, p‐NF‐κB and NLRP3 in the hippocampus after the behavioral study (Figure 6A,B). Compared to naive mice, ASC, Cleaved caspase 1, IL‐18, NF‐κB, p‐NF‐κB and NLRP3 expression increased in the EAE mice. Our findings also revealed that *A. muciniphila* treatment attenuated the increase in ASC, cleaved caspase 1, IL‐18, NF‐κB, p‐NF‐κB and NLRP3, while NLRP3 expression remained unaffected in EAE mice. These findings provide further evidence that *A. muciniphila* treatment can partly mitigate hippocampal NLRP3‐mediated neuroinflammation.

## 4 Discussion

A multitude of investigations have documented shifts in the microbiome of MS patients, including increased levels of *A. muciniphila* and decreased abundance of butyrate‐producing bacteria [8, 31]. Contrary to the prevailing notion that increased *A. muciniphila* may exacerbate MS pathology, studies have shown that *A. muciniphila* isolated from MS patients actually mitigated EAE, associated with a decline in retinoid‐related orphan nuclear receptor γt (RORγt^+^) and IL17‐producing γδ T cells. Nonetheless, limited literature examines the impact of probiotic *A. muciniphila* on neuroimmune function and cognition [4, 5]. Herein, our investigation unveils that *A. muciniphila* pre‐treatment ameliorates EAE pathology (reducing axonal damage, demyelination, and inflammation) and dampens Th17 response. Analysis via 16S rRNA sequencing demonstrates that *A. muciniphila* enhances gut bacterial diversity while decreasing the F/B ratio. Furthermore, *A. muciniphila* treatment diminishes IL17‐producing T cells and promotes Tregs in the SILP of healthy mice, altering intestinal barrier integrity. Remarkably, *A. muciniphila* treatment may exhibit anxiolytic effects and mitigate cognitive impairment by suppressing hippocampal NLRP3‐mediated neuroinflammation. These findings collectively indicate that *A. muciniphila* ‐induced alterations in the gut microbiota may partly prevent central nervous system immunomodulatory responses and cognitive deficits.

Accumulating data have demonstrated the potential of *A. muciniphila* in mitigating metabolic syndrome and intestinal mucosal damage through its capacity to induce an anti‐inflammatory response and regulate intestinal homeostasis [32]. Elevated *F/B* ratios have been strongly associated with the pro‐inflammatory milieu and immunological dysregulation features of autoimmune diseases [33], suggesting a potential mechanism contributing to the amelioration of EAE. Our investigation revealed that supplementation with *A. muciniphila* led to an obvious elevation in both the diversity and abundance of gut microbiota, accompanied by a decrease in the *F/B* ratio at the phylum level. Additionally, *A. muciniphila* supplementation increased the abundance of *Verrucomicrobia*, a phylum often recognized for its probiotic properties and ability to attenuate inflammatory immune responses [34].

The gut microbiota exerts multifaceted control over immune responses, impacting antigen presentation, cytokine production, and lymphocyte function [35]. Herein, we assessed the effect of *A. muciniphila* treatment on T cell responses, crucial for regulating and perpetuating encephalitogenic immune damage [6, 7]. Our findings demonstrated that *A. muciniphila* treatment mitigated Th17 cell infiltration into the CNS and LN. Th17 and Treg, two distinct T cell subsets with inherent plasticity, undergo differentiation influenced by the surrounding microenvironment [36]. Interestingly, we observed no significant alterations in the percentages of LN Treg cells following *A. muciniphila* treatment, indicating that the Treg response in CNS and LN may remain unaffected in this experimental context. Furthermore, the equilibrium between Treg and Th17 cells holds pivotal importance in the pathogenesis of gut inflammation [37]. Notably, the diminished gut microbiota capacity to metabolize ethanolamine triggers glucose metabolic dysfunction, inflammation, and intestinal permeability, whereas restoring ethanolamine‐metabolizing ability via new probiotic therapy can reverse these abnormalities [38]. Our investigation revealed an imbalance in the Th17/Treg ratio in EAE mice, which was effectively rectified by *A. muciniphila* treatment. Given the abundant presence of T cells in the SILP, *A. muciniphila* treatment conferred reduced susceptibility to disruption of the intestinal mucosal barrier. Consequently, the attenuated permeability of the gut barrier and blood–brain barrier (BBB) might contribute to the diminished infiltration of pathological T cells into the CNS and bloodstream.

Emerging research has consistently demonstrated a correlation between dysbiosis and psychological conditions such as depression and cognitive decline, as evidenced by alterations in the gut microbial compositions of Alzheimer's disease and major depressive disorder patients when compared to healthy individuals. These changes encompass variations in microbial diversity and the relative abundance of specific bacterial taxa [39, 40]. Our previous investigations have further elucidated the impact of fecal microbiota transplantation from autoimmune encephalitis patients on modulating Th17 responses and associated behaviors in murine models [9]. Additionally, the experimental EAE mouse model effectively mirrors the neuroinflammatory and behavioral characteristics observed in relapsing–remitting MS (RRMS), including symptoms reminiscent of depression and anxiety [41]. Remarkably, treatment with *A. muciniphila* has shown no obvious anti‐anxiety effects but partly improves cognitive impairment in EAE mice, underscoring its potential role in enhancing cognitive function. These observations support the involvement of *A. muciniphila* in conferring cognitive benefits. Harnessing probiotic *A. muciniphila* to regulate the gut microbiome presents a promising route for modulating immune responses. However, it is imperative to carefully consider factors such as timing, dosage, and duration of gut microbiota therapy with *A. muciniphila* , as these variables may yield divergent outcomes.

The molecular mechanisms of cognition protection underlying the protective effect of *A. muciniphila* may be associated with NLRP3‐mediated neuroinflammation. We found that *A. muciniphila* treatment inhibited hippocampal NLRP3‐mediated ASC, Cleaved Caspase 1, IL‐18, NF‐κB, p‐NF‐κB and NLRP3, while leaving NLRP3 expression unaffected. The NLRP3 inflammasome represents a critical player in neuroinflammatory processes and is implicated in the pathogenesis of both the intestine and brain [42]. Notably, severe memory deficits manifest in the later phases of EAE, and cognitive impairments were mitigated by the administration of the NLRP3 inflammasome inhibitor MCC950. Furthermore, IL18, a downstream effector of the NLRP3 inflammasome, has been shown to induce A1‐type reactive astrocytes, impairing hippocampal neurons via the secretion of cleaved caspase‐3 [43]. Collectively, these findings suggest that *A. muciniphila* ‐mediated cognitive protection may be intricately linked to alterations in the gut microbiota.

In summary, our results underscore the neuroprotective and partly cognition‐enhancing effects of *A. muciniphila* in EAE through microbiota modulation, resulting in the amelioration of clinical symptoms, restoration of the Th17/Treg balance, and suppression of hippocampal NLRP3‐mediated neuroinflammation. This research implies the significant role of the same bacterium as a double‐edged sword in varying immune environments within the host. Considering the therapeutic and preventive potentials of probiotics in managing autoimmune diseases, our study provides a scientific basis for elucidating the mechanisms underlying *A. muciniphila* 's action via the microbiota‐gut‐brain axis, offering insights into new therapeutic strategies.

## Author Contributions

H.C. and D.H. designed the experiments. X.L., D.L., X.H., X.S., and W.H. performed the experiments. H.C. and D.L. drafted the manuscript. X.C., Y.X., and X.W. revised the manuscript. All authors have participated in the review and editing.

## Ethics Statement

This study was approved by the Medical Ethics Committee of the First Affiliated Hospital of Nanchang University (approval ID: 2021‐9024).

## Conflicts of Interest

The authors declare no conflicts of interest.

## Supporting information


Data S1.

## Data Availability

The data sets generated during and/or analyzed during the current study are available from the corresponding authors upon reasonable request.
