# Supplementary material for: Akkermansia muciniphila Modulates Central Nervous System Autoimmune Response and Cognitive Impairment by Inhibiting Hippocampal NLRP3‐Mediated Neuroinflammation
Source: CNS Neurosci Ther. 2025 Mar 6;31(3):e70320. doi: 10.1111/cns.70320 (PMC11884925; doi:10.1111/cns.70320)

Supplemental Figure: Uncropped western blot images

Supplemental Figure related to Figure 4A

Claudin-5, 22kDa

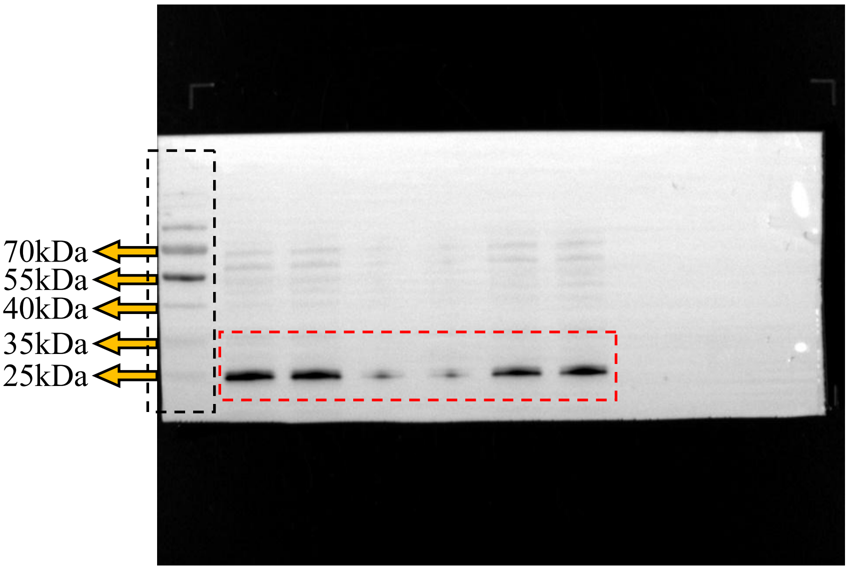

Occludin, 65kDa

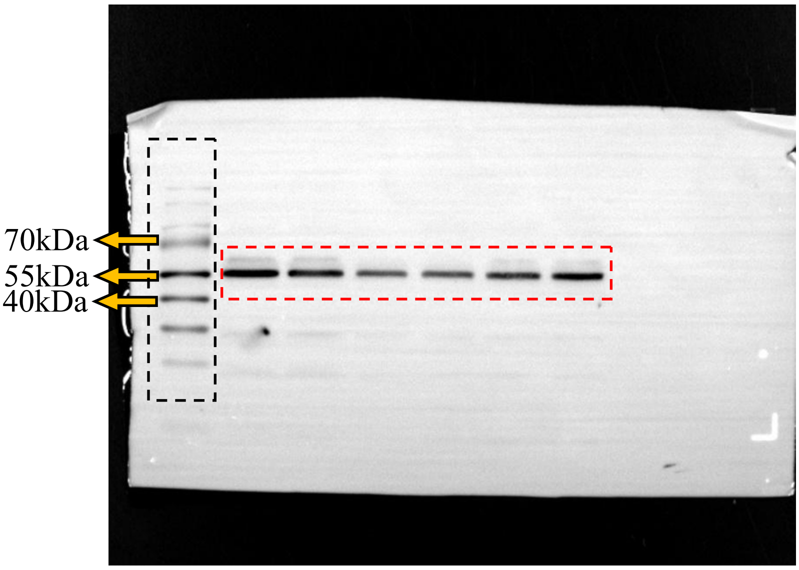

JAM-A, 65kDa

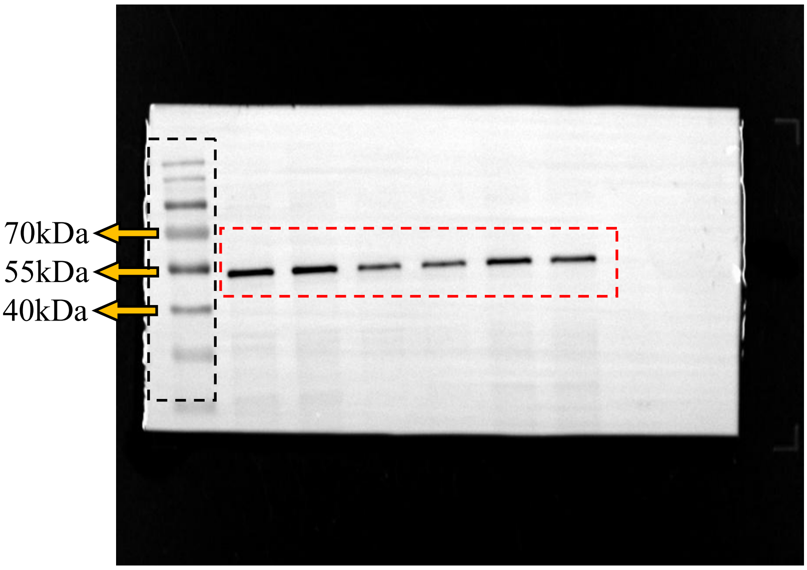

E-Cadherin, 70kDa

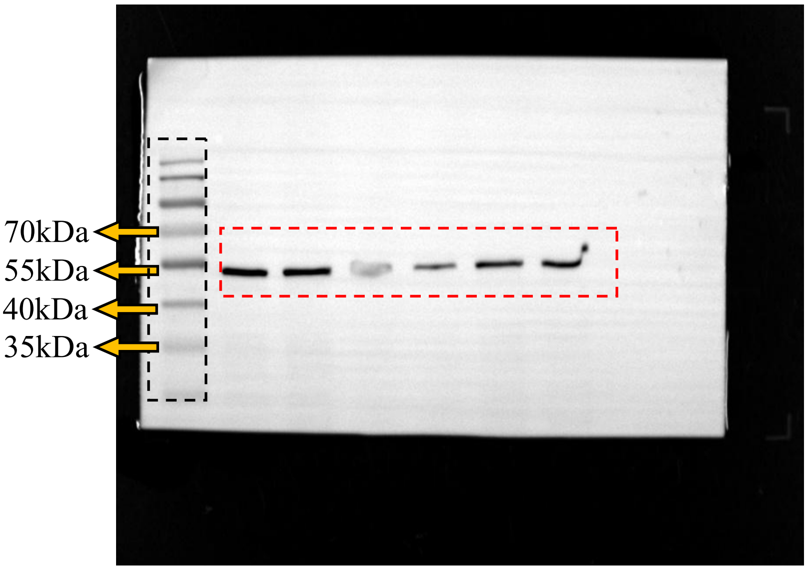

GAPDH, 37kDa

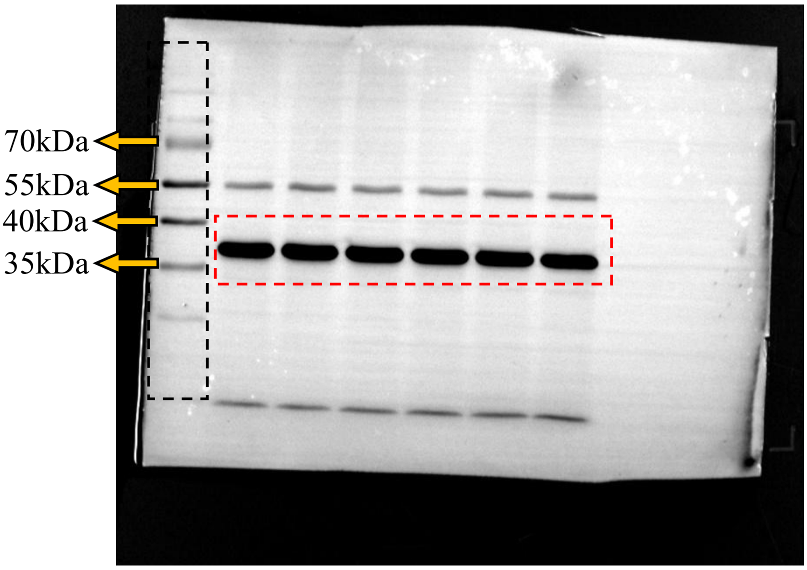

Supplemental Figure 6A

Cleaved Caspase 1, 22kDa

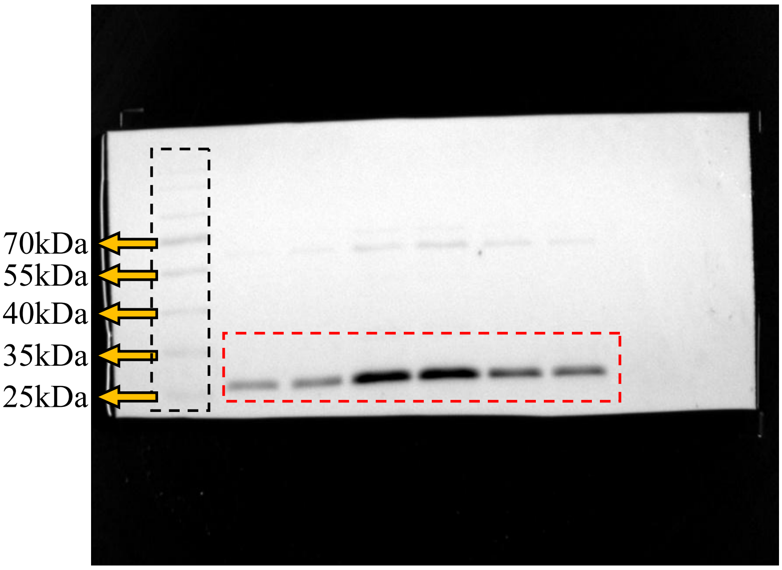

IL-18, 23kDa

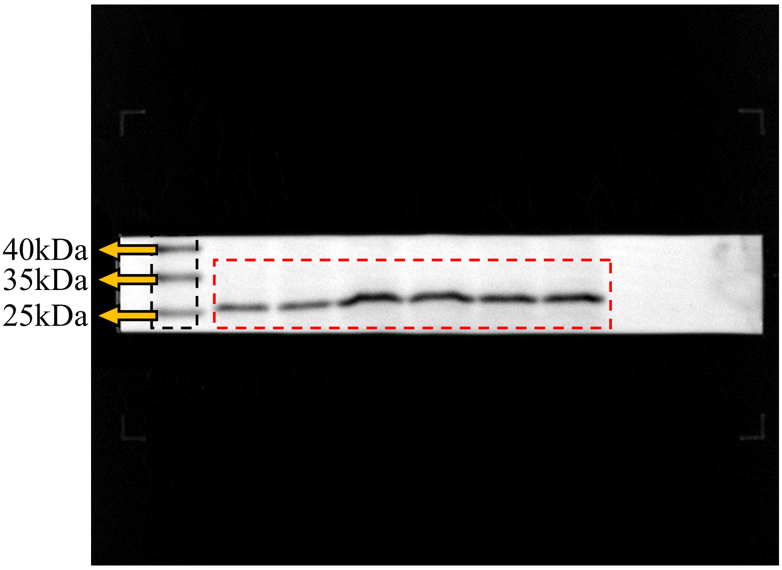

ASC, 37kDa

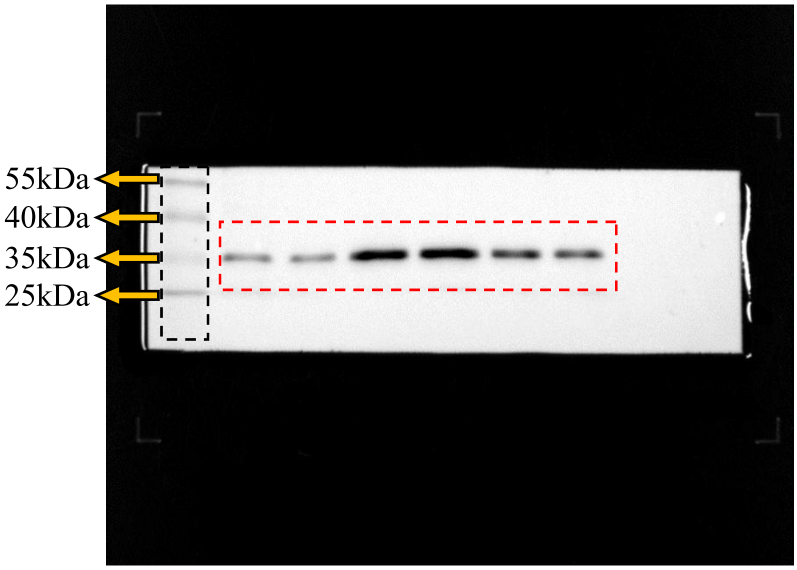

NF-κβ, 65kDa

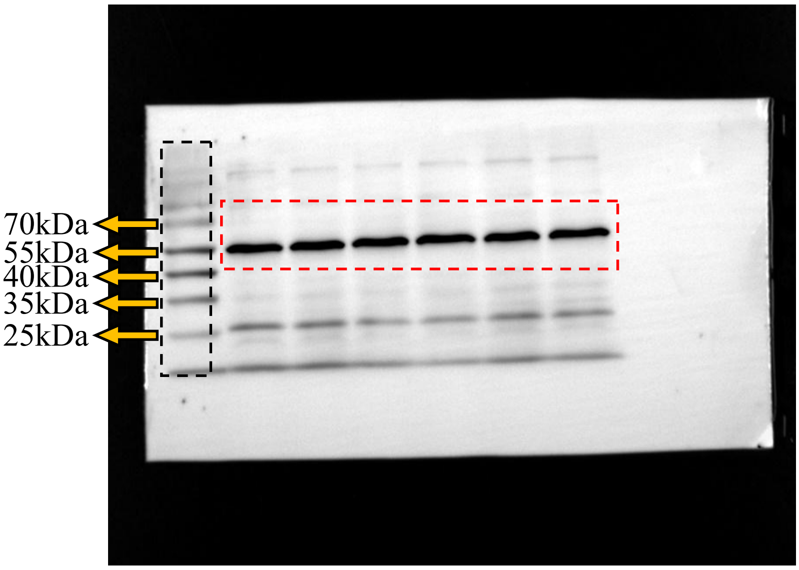

p-NF-κβ, 65kDa

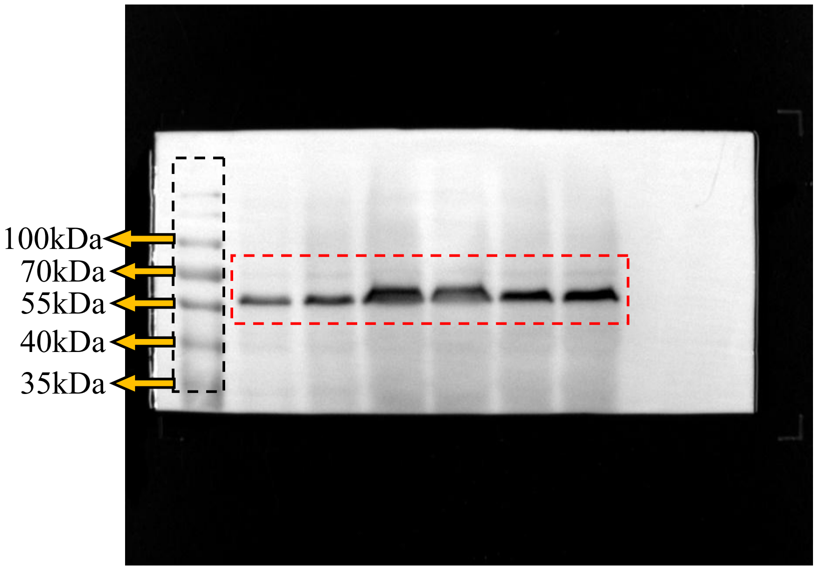

NLRP3, 90kDa

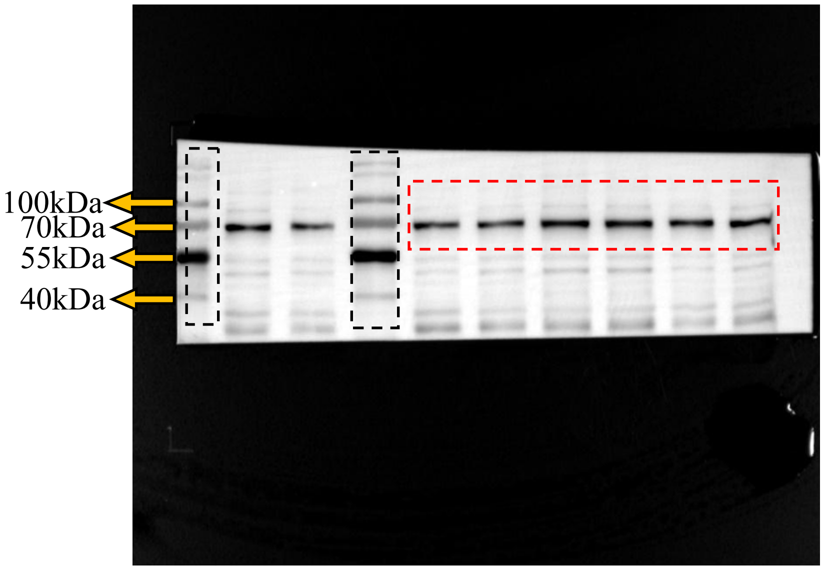

GAPDH, 37kDa

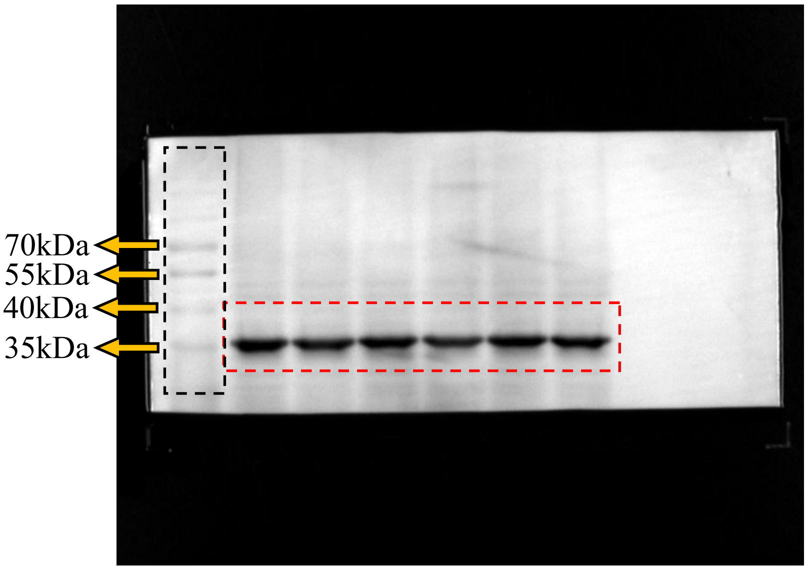

Supplement: Supplementary file 1 — Data S1. [file CNS-31-e70320-s001.zip › cns70320-sup-0002-Supinfo.pdf]
