# Supplementary material for: Akkermansia muciniphila Modulates Central Nervous System Autoimmune Response and Cognitive Impairment by Inhibiting Hippocampal NLRP3‐Mediated Neuroinflammation
Source: CNS Neurosci Ther. 2025 Mar 6;31(3):e70320. doi: 10.1111/cns.70320 (PMC11884925; doi:10.1111/cns.70320)
Supplement: Supplementary file 1 — Data S1. [file CNS-31-e70320-s001.zip › cns70320-sup-0001-Figures.doc]

**Open-field test.** Totle distance of the three groups. Mean±SEM. ns, not significant. **p<0.01.

**Novel object recognition test.** Mean speed and discrimination index of the three groups. Mean±SEM. *p<0.05, **p<0.01.
